# PDLIM1, a novel miR-3940-5p target, regulates the malignant progression of diffuse large B-cell lymphoma

**DOI:** 10.1080/15384047.2024.2429175

**Published:** 2024-11-20

**Authors:** Jinfeng Zhu, Huifang Xiao, Chuntuan Li, Xiaofeng Li, Jinyang Zheng, Xihu Yao, Shaoxiong Wang, Xiongpeng Zhu

**Affiliations:** a Department of Oncology, Quanzhou First Hospital Affiliated to Fujian Medical University, Quanzhou, China; b Department of Hematology, Quanzhou First Hospital Affiliated to Fujian Medical University, Quanzhou, Fujian Province, China; c Department of Pathology, Quanzhou First Hospital Affiliated to Fujian Medical University, Quanzhou, Fujian Province, China

**Keywords:** PDLIM1, miR-3940-5p, diffuse large B-cell lymphoma, proliferation, apoptosis

## Abstract

**Background:**

PDZ And LIM domain protein 1 (PDLIM1), a protein-coding gene, has been widely reported to exhibit differential expression patterns across various human cancers, including hematological malignancies. This study aimed to investigate PDLIM1 expression pattern and its functional role in diffuse large B-cell lymphoma (DLBCL) both *in vitro* and *in vivo*.

**Methods:**

PDLIM1 expression patterns were reanalyzed using data from the Gene Expression Omnibus, and the results were subsequently validated in patient tissue samples and a panel of four DLBCL cell lines. MicroRNA-3940-5p (miR-3940-5p) was identified as an upstream regulator of PDLIM1. The interaction between PDLIM1 and miR-3940-5p and its effects on DLBCL cellular activities and cancer development were further explored using a DLBCL mouse model.

**Results:**

Elevated PDLIM1 expression was observed in DLBCL cells and tissues. Reduced cell proliferation and increased DLBCL cell apoptosis were observed following the knockdown of this gene. Furthermore, short hairpin RNA (shRNA)-mediated PDLIM1 knockdown diminished tumorigenesis of DLBCL cells in nude mice. miR-3940-5p was identified as an upstream regulator of PDLIM1. PDLIM1 expression and function were negatively modulated by the upregulation of miR-3940-5p, consequently affecting the malignant phenotype of DLBCL cells.

**Conclusion:**

These findings suggest that the miR-3940-5p/PDLIM1 axis may play a crucial role in DLBCL pathogenesis and could potentially be exploited for therapeutic interventions.

## 1. Introduction

Diffuse large B-cell lymphoma (DLBCL) is a prevalent subtype of non-Hodgkin lymphoma worldwide, accounting for 25 to 30% of cases.^1,2^ While DLBCL can occur during childhood, its incidence increases with age, with the majority of cases diagnosed in individuals over 60 years. Despite being an aggressive condition, DLBCL is typically identified at an advanced stage.^1^ Comprehensive analyses of molecular aberrations are crucial for refining disease classification and developing innovative therapeutic approaches.

PDZ and LIM domain protein 1 (PDLIM1) is a protein-coding gene exhibiting differential expression across various human cancers, including diffuse large B-cell lymphoma (DLBCL).^3–8^ The deregulated expression of PDLIM1 has been linked to proliferation, metastasis, and survival of malignant cells.^3^ MicroRNAs (miRNAs) are non-coding RNAs (ncRNAs) that regulate post-transcriptional gene expression. miRNAs primarily interact with the 3′-untranslated region (3′-UTR) of target mRNAs, inducing translation suppression and mRNA degradation.^9^ miRNAs have been implicated in diverse cancer biological processes, including growth, migration, invasion, angiogenesis, and apoptosis.^9,10^ However, our understanding of miRNA – mRNA interactions as regulatory module remains limited.^11^ Particularly, the specific miRNA regulators for PDLIM1 in DLBCL remain unknown. Therefore, this study aimed to investigate PDLIM1 expression and function in DLBCL both *in vivo* and *in vitro*, identify its upstream miRNA regulators, and evaluate the impact of their interactions on the aggressiveness of DLBCL cells.

## 2. Results

### 2.1. PDLIM1 is highly expressed in DLBCL tissues

**Figure 1.**
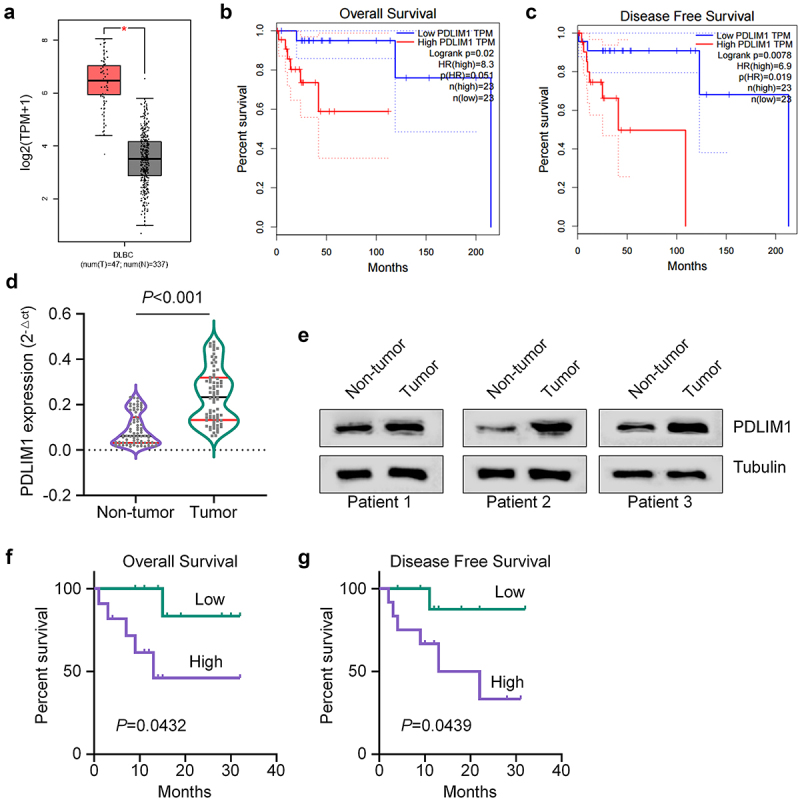
PDLIM1 is highly expressed in DLBCL tissues. (a) PDLIM1 expression levels in DLBCL samples compared to normal tissues, based on GEPIA analysis of the TCGA data. (b-c) Kaplan-Meier survival curves showing overall survival (b) and disease-free survival (c) for DLBCL patients with high vs. low PDLIM1 expression from TCGA data. (d) qRT-pcr analysis of PDLIM1 mRNA levels in 76 DLBCL tumor samples compared to matched non-carcinoma samples. (e) Western blot analysis of PDLIM1 protein levels in three pairs of DLBCL tumor and adjacent non-tumor tissues. (f–g) Kaplan-Meier survival curves showing overall survival (f) and disease-free survival (g) for DLBCL patients stratified by PDLIM1 expression levels.

We initiated our investigation by conducting gene expression analyses using Gene Expression Profiling Interactive Analysis (GEPIA; http://gepia.cancer-pku.cn/),^12^ an interactive web application designed to analyze gene expression levels in samples from The Cancer Genome Atlas (TCGA).^13^ Our analysis revealed that PDLIM1 exhibited high expression within Diffuse Large B-cell Lymphoma (DLBCL) samples (Figure 1a). Furthermore, we observed that DLBCL cases with PDLIM1 up-regulation were associated with poor overall survival (OS) (Figure 1b) and disease-free survival (DFS) compared to cases with PDLIM1 down-regulation (Figure 1c). To validate these findings, we proceeded to assess PDLIM1 expression and its correlation with the clinicopathological features in DLBCL patients. We obtained 76 DLBCL and 76 matched non-carcinoma clinical samples from DLBCL cases., and the PDLIM1 levels were quantified using real-time quantitative PCR (qRT-PCR). The results showed significantly increased PDLIM1 expression in tumor samples compared to non-carcinoma samples (Figure 1d). To further confirm these findings at the protein level, we performed western blot analysis on three pairs of samples. Consistent with the mRNA results, PDLIM1 protein levels were elevated in DLBCL samples relative to non-carcinoma samples (Figure 1e). Patients were then stratified into high- and low-expression groups (high or low; *n* = 38 per group) based on the median expression value of PDLIM1 in the tumor samples determined by qRT-PCR. The correlation analysis demonstrated that PDLIM1 expression correlated with B symptoms, tumor Ann Arbor stage, and international prognostic index score (Table 1). Kaplan-Meier plotter analysis further revealed that in DLBCL patients, high PDLIM1 expression predicted lower OS and DFS compared to low PDLIM1 expression (Figure 1f,g). Taken together, these findings provide strong evidence that PDLIM1 is up-regulated in DLBCL tissues and its expression levels correlate with poor clinical outcomes.

**Table 1.** Correlations of PDLIM1 expression with clinicopathologic features of diffuse large B-cell lymphoma.

Variable	PDLIM1 expression		*p-*value
	Low (*n* = 38)	High (*n* = 38)	
Mean age(years)	51.62 ± 11.54	57.29 ± 17.37	0.1173
Sex			0.2512
Female	16	21	
Male	22	17	
B-symptoms			0.0336
No	19	10	
Yes	19	28	
Performance score			0.1996
0 + 1	30	25	
2-3	8	13	
Stage			0.0171
I – II	14	5	
III – IV	24	33	
IPI score			0.0136
0–2	17	7	
3–5	21	31	
S-LDH			0.1687
Normal	22	16	
Elevated	16	22	
Extranodal foci			0.0661
No	22	14	
Yes	16	24	
Splenomegaly			0.0114
No	32	22	
Yes	6	16	

### 2.2. PDLIM1 silencing suppresses DLBCL cell growth and induces their apoptosis

**Figure 2.**
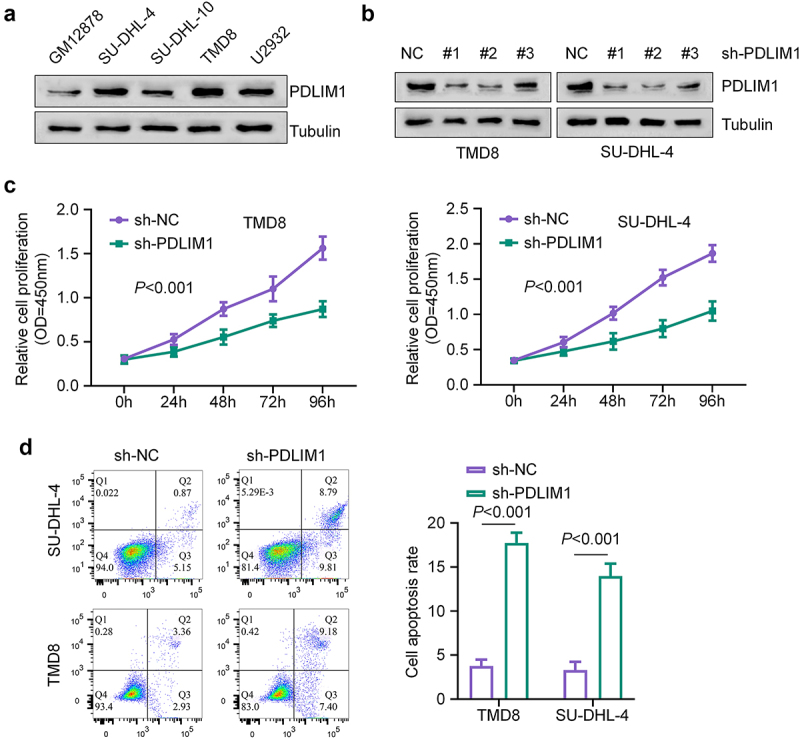
PDLIM1 silencing suppresses DLBCL cell growth and induces their apoptosis. (a) Western blot analysis of PDLIM1 protein levels in four DLBCL cell lines compared to normal human B lymphocytes (GM12878). (b) Western blot confirmation of PDLIM1 knockdown efficiency using different shRNAs in TMD8 and SU-DHL-4 cells. (c) Cell proliferation analysis (CCK-8 assay) for TMD8 and SU-DHL-4 cells following PDLIM1 knockdown. (d) Apoptosis analysis (Annexin V and PI staining) of TMD8 and SU-DHL-4 cells after PDLIM1 knockdown.

To further investigate the role of PDLIM1 in regulating the malignancy of DLBCL cells, we collected four DLBCL cell lines: SU-DHL-4, SU-DHL-10, TMD8, and U2932. We examined the expression of PDLIM1 in these cells, comparing them to normal human B lymphocytes (GM12878). Our results revealed that PDLIM1 protein levels were elevated in all DLBCL cell lines relative to healthy human B lymphocytes, with SU-DHL-4 and TMD8 showing the highest expression levels (Figure 2a). To elucidate the functional significance of PDLIM1, we established stable knockdown of PDLIM1 using three different shRNAs: sh-PDLIM1 #1, sh-PDLIM1 #2, and sh-PDLIM1 #3. Among these constructs, sh-PDLIM1 #2 showed the highest knockdown efficiency and was therefore selected for subsequent experiments, henceforth referred to as sh-PDLIM1. We then investigated the effects of PDLIM1 knockdown on cell viability and survival in TMD8 and SU-DHL-4 cells. Our findings revealed that knockdown of PDLIM1 significantly decreased cell viability and promoted apoptosis in both cell lines (Figure 2c,d). These results collectively demonstrate that silencing PDLIM1 has a profound impact on DLBCL cells *in vitro*, manifesting as inhibition of proliferation and induction of apoptosis. This suggests that PDLIM1 may play a crucial role in the survival and proliferation of DLBCL cells.

### 2.3. *Knockdown of PDLIM1 inhibits tumor growth of DLBCL cells* in vivo

**Figure 3.**
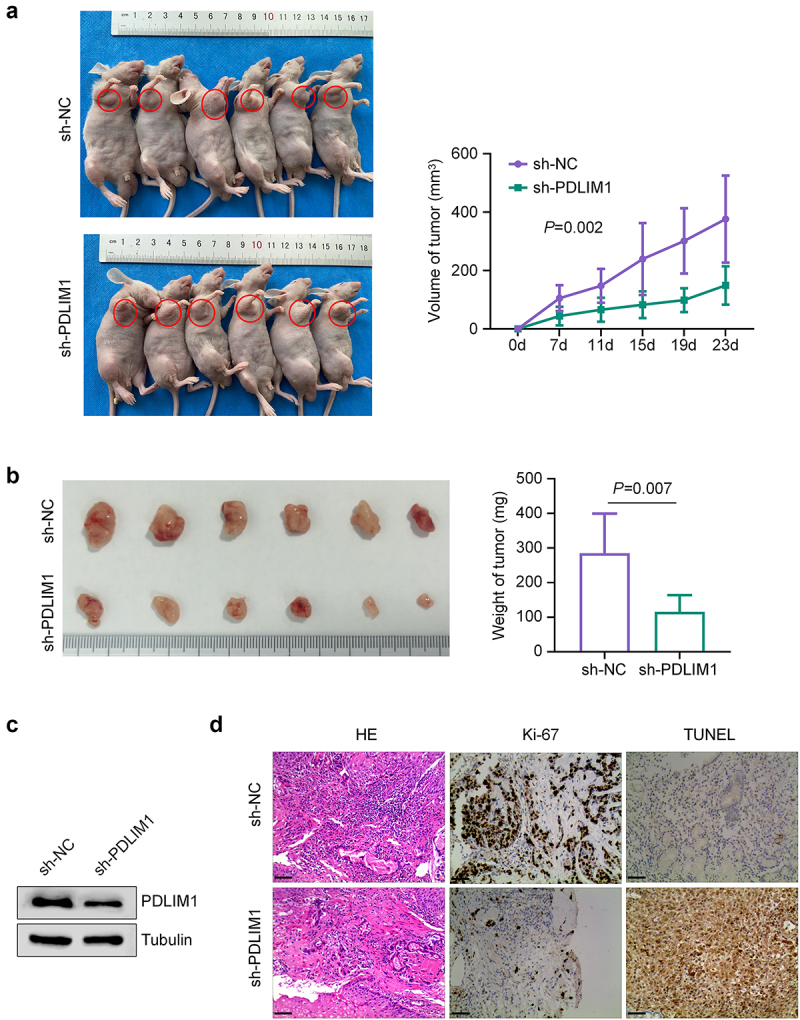
Knockdown of PDLIM1 inhibits tumor growth of DLBCL cells *in vivo*. (a) tumor growth curves of xenografts derived from SU-DHL-4 cells expressing sh-PDLIM1 or sh-nc. (b) Final tumor weights at the end of the experiment. The images of tumor samples in each group was shown. (c) Western blot analysis of PDLIM1 expression in tumor samples from sh-PDLIM1 and sh-nc groups. (d) H&E staining, ki-67 immunohistochemistry, and TUNEL assay results for tumor tissues from sh-PDLIM1 and sh-nc groups.

To investigate the *in vivo* effects of PDLIM1 knockdown, we established a nude mouse xenograft model with SU-DHL-4 cells stably expressing sh-PDLIM1 or sh-NC (negative control). Tumor growth was monitored by measuring tumor volume every seven days from Day 7 to Day 35, with final tumor weight recorded at the end of the experiment. PDLIM1 silencing significantly suppressed tumor growth compared to the control group (Figure 3a,b). Western blot analysis confirmed reduced PDLIM1 expression in tumor samples from the PDLIM1 knockdown group (Figure 3c). To further characterize the effects of PDLIM1 knockdown on cell proliferation and survival in the tumor tissues, we performed Hematoxylin and eosin (H&E) staining, immunohistochemical staining for Ki-67 (a well-established proliferation marker), and terminal deoxynucleotidyl transferase (TdT)-mediated dUTP-biotin nick end labeling (TUNEL) for apoptotic event evaluation. Our histological analyses revealed significant differences between the sh-PDLIM1 and control groups. We observed a marked reduction in Ki-67-positive cells in the sh-PDLIM1 group, indicating decreased proliferation. Conversely, the number of apoptotic cells, as visualized by TUNEL staining, was significantly elevated in the sh-PDLIM1 group (Figure 3d). These *in vivo* findings corroborate our *in vitro* results, providing compelling evidence for the inhibitory effect of PDLIM1 knockdown on DLBCL cell proliferation. Furthermore, PDLIM1 silencing not only reduces tumor growth but also promotes apoptosis in DLBCL tumors, highlighting its potential as a therapeutic target.

### 2.4. PDLIM1 is a potential target of miR-3940-5p

**Figure 4.**
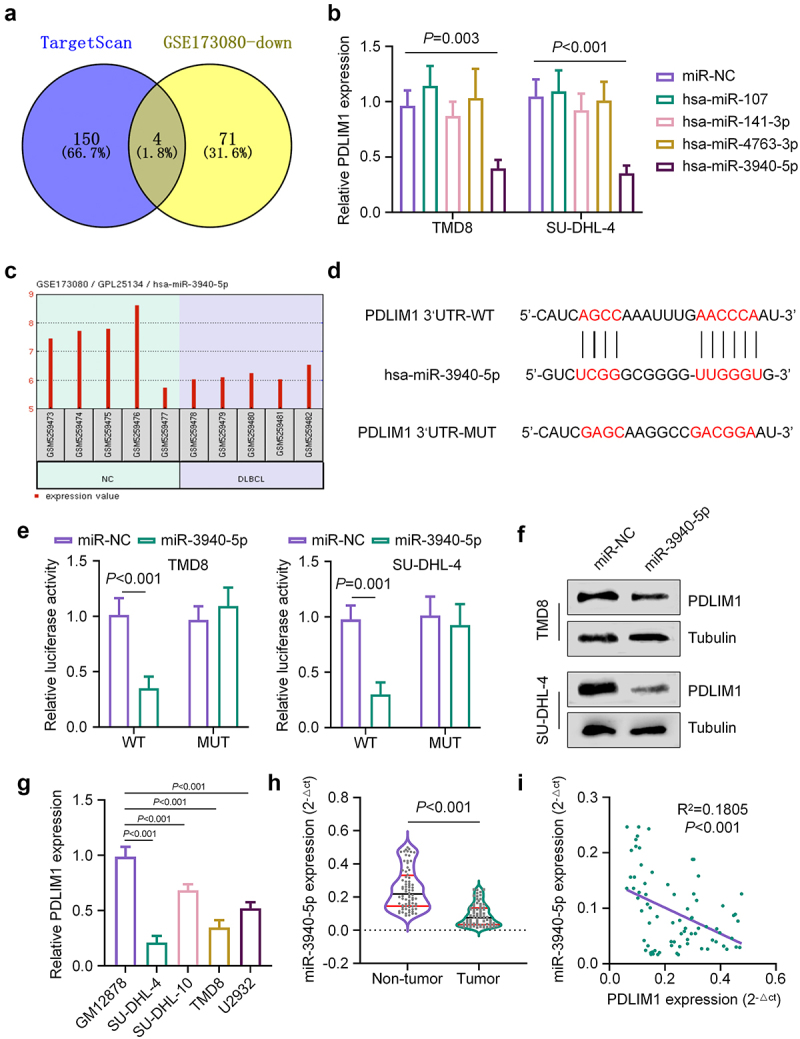
PDLIM1 is a potential target of miR-3940-5p. (a) venn diagram showing the intersection of predicted miRNAs targeting PDLIM1 and downregulated miRNAs in DLBCL. (b) qRT-pcr analysis of PDLIM1 mRNA levels in TMD8 and SU-DHL-4 cells transfected with mimics of the four identified miRnas. (c) miR-3940-5p expression levels in DLBCL samples from GSE173080 dataset. (d) Predicted binding sites of miR-3940-5p on PDLIM1 mRNA 3‘UTR. (e) Luciferase reporter assay results for wild-type and mutated PDLIM1 3‘UTR reporters in the presence of miRNA mimic or miR-nc (negative control). (f) Western blot analysis of PDLIM1 protein levels in TMD8 and SU-DHL-4 cells transfected with miR-nc or miR-3940-5p mimic. (g) qRT-pcr analysis of miR-3940-5p expression in DLBCL cell lines compared to normal human B lymphocytes. (h) qRT-pcr analysis of miR-3940-5p levels in 76 DLBCL samples and matched non-carcinoma samples. (i) Correlation analysis between PDLIM1 and miR-3940-5p expression levels in DLBCL samples.

To identify potential miRNAs that regulate PDLIM1 in DLBCL, we employed a dual approach combining computational prediction and expression analysis. Computational prediction using TargetScan resources (https://www.targetscan.org/vert_80/) identified 154 miRNAs that might interact with PDLIM1. Concurrently, we analyzed GSE173080 microarray data from the Gene Expression Omnibus (GEO) and identified 75 miRNAs with reduced expression in DLBCL. The intersection of these two approaches revealed four miRNAs: hsa-miR-107, hsa-miR-141-3p, hsa-miR-4763-3p, and hsa-miR-3940-5p (Figure 4a).

To validate these findings, we treated TMD8 and SU-DHL-4 cells with mimics of the four identified miRNAs and assessed PDLIM1 mRNA expression post-transfection. Notably, only miR-3940-5p-treated cells exhibited significantly reduced expression of PDLIM1 mRNA (Figure 4b). This result aligns with the GSE173080 data, which showed decreased miR-3940-5p expression in DLBCL samples (Figure 4c). Further investigation into the interaction between miR-3940-5p and PDLIM1 revealed potential binding sites on the 3‘UTR of PDLIM1 mRNA (Figure 4d). To confirm this interaction, we conducted a luciferase reporter assay using the wild-type (WT) PDLIM1 3‘UTR reporter or the mutated (MUT) reporter. Overexpression of miR-3940-5p significantly reduced luciferase activity in cells transfected with the WT reporter, while no difference was observed in the mutated reporter (Figure 4e), suggesting an interaction between miR-3940-5p and the PDLIM1 3‘UTR. Western blot analysis of PDLIM1 protein levels in TMD8 and SU-DHL-4 cells transfected with miR-NC (negative control) or miR-3940-5p mimic further supported these findings. Upregulation of miR-3940-5p led to a decrease in PDLIM1 protein levels (Figure 4f). To establish the clinical relevance of these findings, we examined miR-3940-5p expression in various cell lines and patient samples. All tested DLBCL cell lines showed lower miR-3940-5p expression compared to normal human B lymphocytes (GM12878) (Figure 4g). Moreover, analysis of 76 DLBCL samples and 76 matched non-carcinoma samples revealed significantly reduced miR-3940-5p levels in tumor samples compared to control samples (Figure 4h). Importantly, we observed a negative correlation between PDLIM1 and miR-3940-5p levels in these samples (Figure 4i), further supporting the regulatory relationship between miR-3940-5p and PDLIM1. Collectively, these findings provide strong evidence that PDLIM1 is a target gene of miR-3940-5p, and suggest that the downregulation of miR-3940-5p in DLBCL may contribute to the overexpression of PDLIM1 in DLBCL pathogenesis.

### 2.5. miR-3940-5p overexpression inhibits DLBCL cell growth and induces apoptosis by targeting PDLIM1

**Figure 5.**
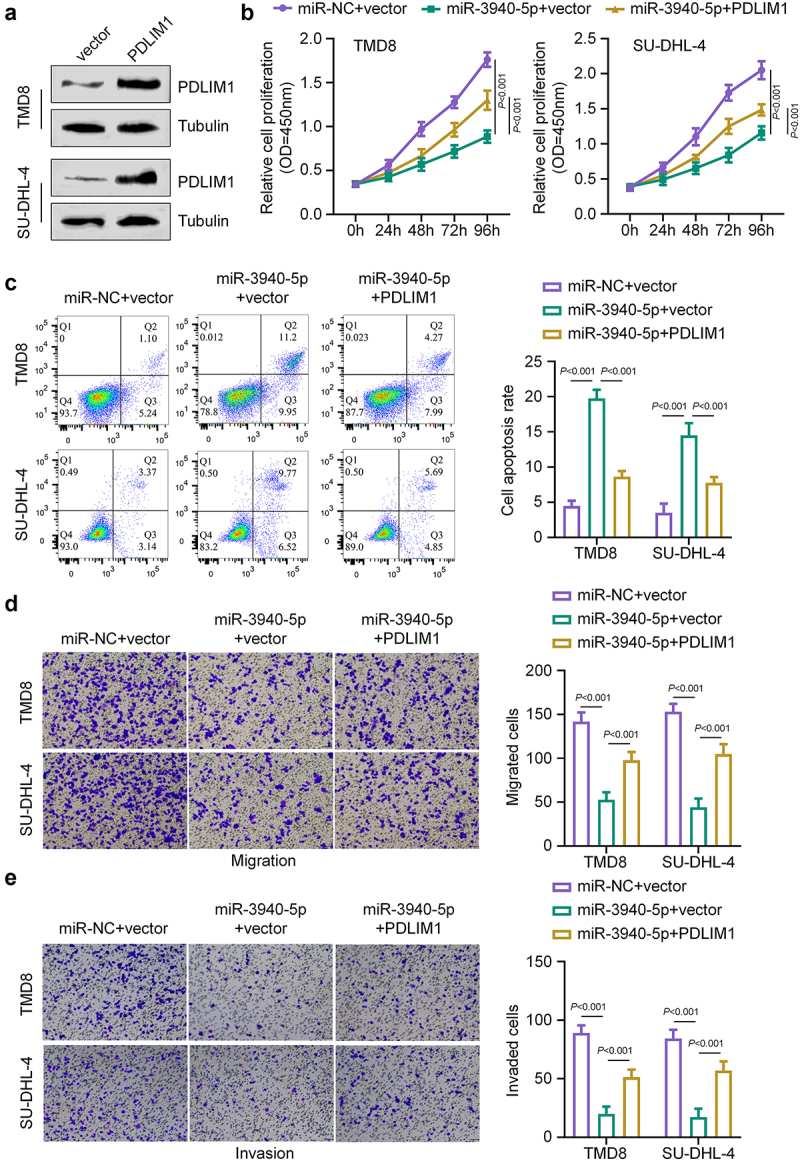
miR-3940-5p overexpression inhibits DLBCL cell growth and induces apoptosis by targeting PDLIM1. (a) Western blot confirmation of PDLIM1 overexpression in SU-DHL-4 and TMD8 cells. (b) Cell proliferation assay results for SU-DHL-4 and TMD8 cells transfected with miR-NC+vector, miR-3940-5p+vector, or miR-3940-5p+PDLIM1 expression vector. (c) Apoptosis analysis of SU-DHL-4 and TMD8 cells under different transfection conditions. (d) Transwell migration assay results showing cell migration under different transfection conditions. (e) Transwell invasion assay results showing cell invasion under different transfection conditions.

To investigate the functional consequences of the miR-3940-5p and PDLIM1 interaction in DLBCL, we transfected DLBCL cells with three different combinations: miR-NC+vector (control), miR-3940-5p+vector, or miR-3940-5p+PDLIM1 expression vector. Western blot analysis confirmed increased PDLIM1 expression in PDLIM1-transfected SU-DHL-4 and TMD8 cells compared to vector-transfected controls (Figure 5a). Cell proliferation analysis showed that cells in the miR-3940-5p+vector group exhibited significantly decreased cell growth capacity compared to the miR-NC+vector group (Figure 5b. Importantly, concurrent overexpression of miR-3940-5p and PDLIM1 (miR-3940-5p+PDLIM1 group) partially rescued this effect, resulting in higher levels of cell proliferation in both SU-DHL-4 and TMD8 cells compared to the miR-3940-5p+vector group (Figure 5b). Meanwhile, the apoptosis rate was significantly increased in miR-3940-5p+vector transfected SU-DHL-4 and TMD8 cells compared to those transfected with miR-NC+vector, an effect that was partially suppressed by PDLIM1 overexpression (Figure 5c). Transwell migration assay revealed that miR-3940-5p overexpression significantly reduced cell migration, while PDLIM1 co-expression partially restored this ability (Figure 5d). Similarly, miR-3940-5p also suppressed cell invasion in the Transwell invasion assay, an effect partially reversed by PDLIM1 overexpression (Figure 5e). These findings collectively suggest that miR-3940-5p exerts its tumor-suppressive effects in DLBCL cells primarily through the downregulation of PDLIM1.

## 3. Discussion

PDLIM1 has been shown to be differentially expressed in various human tumors, including DLBCL.^3,4^ Functional analyses have revealed that PDLIM1 plays a crucial role in cancer cell proliferation, metastasis, and survival.^3^ In our study, we observed high expression of PDLIM1 in both DLBCL cells and clinical samples, which correlated with increased cell proliferation. Our results further demonstrated that silencing PDLIM1 significantly reduced cell proliferation and increased apoptosis in DLBCL cells. Moreover, we extended our investigation to an *in vivo* mouse model of DLBCL. Notably, mice bearing PDLIM1-knockdown tumors exhibited decreased average tumor size compared to controls, providing strong evidence for the inhibitory effect of PDLIM1 suppression on DLBCL cell proliferation. In an effort to understand the regulatory mechanisms governing PDLIM1 expression, we employed target prediction tools and identified miR-3940-5p as a potential upstream regulator. Subsequent experiments confirmed that upregulation of miR-3940-5p modulated both PDLIM1 expression and function. Consequently, this regulatory axis significantly impacted DLBCL cell growth and survival. These findings collectively highlight the importance of PDLIM1 in DLBCL pathogenesis and reveal a novel miR-3940-5p/PDLIM1 regulatory pathway that could potentially be exploited for therapeutic interventions in DLBCL.

Previous gene-expression profiling studies have reported that PDLIM1 is upregulated in DLBCL.^4,14^ PDLIM1 is a cytoskeletal protein that acts as an adapter to bring other LIM-interacting proteins to the cytoskeleton. It exhibits a widespread distribution in human tissues, including the lung, heart, spleen, and liver. Through interaction with different proteins within diverse tissues, PDLIM1 exerts its functions in a tissue-specific manner.^3^ Furthermore, PDLIM1 has been implicated in a variety of signal transduction pathways affecting cell growth and migration during cancer occurrence and development.^3^ In corroboration with previous studies, our research demonstrated that PDLIM1 has a critical effect on DLBCL cell growth and survival. Importantly, we showed that PDLIM1 can be effectively targeted by shRNA-mediated gene silencing, potentially blocking its carcinogenic activity. These findings highlight the clinical significance of PDLIM1 as a potential therapeutic target for DLBCL treatment. Further research is warranted to fully elucidate the underlying mechanisms by which PDLIM1 regulates the malignancy of DLBCL cells.

miRNAs play critical roles in modulating gene expression levels and mRNA translation.^15^ These non-coding RNAs are involved in numerous biological processes, and their abnormal levels have been associated with various human cancers, including DLBCL.^16–18^ Similar to protein-coding genes, miRNAs can function as either oncogenes or tumor suppressors, depending on the cellular context in which they are expressed. Oncogenic miRNAs often repress tumor-suppressive mRNAs, whereas tumor-suppressor miRNAs downregulate protein-coding oncogenes. According to our findings, miR-3940-5p exhibits tumor-suppressive properties in DLBCL. The loss of this tumor-suppressor miRNA within DLBCL cell lines can lead to the upregulation of oncogenes, such as PDLIM1, which in turn promotes cancer development. Although the precise contribution of miR-3940-5p to DLBCL pathogenesis remains to be fully elucidated, emerging evidence suggests that its expression is altered in this malignancy. Understanding the expression pattern of miR-3940-5p in DLBCL, along with its mechanisms of action in cancer development, may prove valuable in improving DLBCL classification and predicting disease behavior. This knowledge could potentially contribute to the development of more effective diagnostic and therapeutic strategies for DLBCL.

To conclude, our study demonstrated elevated PDLIM1 expression in both DLBCL cells and tissues, which correlated with poor overall survival and disease-free survival in DLBCL patients. Functional analyses revealed that *in vitro* silencing of PDLIM1 significantly decreased cell proliferation and increased apoptosis in DLBCL cell lines. In the mouse model of DLBCL, PDLIM1 knockdown reduced tumor volume and weight, further supporting the notion that PDLIM1 knockdown can effectively suppress tumor growth. Additionally, we identified miR-3940-5p as an upstream regulator of PDLIM1. Our findings suggest that miR-3940-5p and PDLIM1 form a regulatory network that modulates DLBCL cell growth and survival. These results underscore the clinical significance of PDLIM1 in DLBCL. Further investigations are warranted to elucidate the precise expression patterns and functional roles of PDLIM1 in DLBCL, which could potentially lead to novel therapeutic strategies for this aggressive lymphoma.

## 4. Materials and methods

### 4.1. Human DLBCL sample

A total of 76 frozen DLBCL tissue samples and 76 matched non-carcinoma samples were collected from DLBCL patients at Quanzhou First Hospital Affiliated to Fujian Medical University. Tissue samples were collected during surgical procedures, immediately snap-frozen in liquid nitrogen, and subsequently stored at −80°C until further analysis. All samples were collected with informed consent from patients, and the study protocol was approved by the Ethics Committee of Quanzhou First Hospital Affiliated to Fujian Medical University. The use of these clinical specimens for research purposes complied with institutional guidelines and the Declaration of Helsinki.

### 4.2. Cell culture

Four human DLBCL cell lines (SU-DHL-4, SU-DHL-10, TMD8, and U2932) were acquired from the American Type Culture Collection (ATCC, Manassas, VA, USA). Normal human B lymphocytes (GM12878) were procured from Shanghai Hongshun Biotech. Co., Ltd. (Shanghai, China). The cells were cultured in RPMI-1640 medium (Gibco, Thermo Fisher Scientific, Waltham, MA, USA) supplemented with 10% fetal bovine serum (FBS; Gibco) and 1% penicillin-streptomycin (Invitrogen). All cells were maintained in a humidified incubator at 37°C with 5% CO_2_. The culture medium was changed every 2–3 days, and cells were passaged when they reached 80–90% confluence. All cell lines were regularly tested for mycoplasma contamination using a MycoAlert Mycoplasma Detection Kit (Lonza, Basel, Switzerland) and were found to be negative.

### 4.3. Quantitative reverse transcription PCR

RT-PCR was conducted to assess PDLIM1 and miR-3940-5p mRNA expression in DLBCL tissues and a panel of cell lines (SU-DHL-4, SU-DHL-10, TMD8, and U2932). Total RNA was extracted from treated cells and controls using the RNeasy RNA Isolation Kit (QIAGEN) according to the manufacturer’s protocols. RNA content was subsequently analyzed using a spectrophotometer. cDNA was prepared from total RNA (10 ng) obtained from each sample through reverse transcription using a PrimeScript II 1st Strand cDNA Synthesis Kit (TaKaRa). qRT-PCR was carried out using SYBR Green PCR Master Mix (Thermo Fisher Scientific) with specific primers for PDLIM1 and miR-3940-5p. All experiments were conducted in triplicate to ensure reproducibility of results. Gene levels were analyzed by 2^−ΔΔCt^ approach, with GAPDH and U6 being the endogenous controls for mRNA and miRNA, separately. Primer sequences utilized included 5′-CCCAGCAGATAGACCTCCAG-3′ (forward, F), 5′-TCTGAGCTTCCAAGTGTGTCATA-3′ (reverse, R) for PDLIM1; 5′- CGGTGGGTTGGGGCGG-3′ (F), 5′-AGTGCAGGGTCCGAGGTATT-3′ (R) for miR-3940-5p; 5′- GCACCGTCAAGGCTGAGAAC-3′ (F), 5′ TGGTGAAGACGCCAGTGGA-3′ (R) for GAPDH; and 5′-GCTTCGGCAGCACATATACTAAAAT-3′ (F), 5′ CGCTTCACGAATTTGCGTGTCAT-3′ (R) for U6.

### 4.4. Western-blotting (WB) assay

Total cellular proteins were extracted using a cell lysis buffer (Invitrogen), and protein concentrations were subsequently quantified using a bicinchoninic acid kit (Pierce). Proteins were separated by electrophoresis on SDS-containing polyacrylamide gels and transferred to polyvinylidene fluoride (PVDF) membranes (Millipore). The membranes were blocked with 5% BSA in TBST buffer and then incubated overnight at 4°C with primary antibodies against PDLIM1 (ab45162, Abcam, 1:1000 dilution) and Tubulin (loading control) (ab7291, Abcam, 1:5000 dilution). Following incubation, the membranes were washed with TBST buffer for 10 minutes and then incubated with appropriate horseradish peroxidase (HRP)-labeled secondary antibodies (anti-rabbit IgG, ab205718, Abcam, 1:5000 dilution; anti-mouse IgG, ab205719, Abcam, 1:5000 dilution) for 2 hours at room temperature. After three washes with TBST buffer, the membranes were incubated with SuperSignal West Pico PLUS Chemiluminescent Substrate (Thermo Fisher Scientific, catalog number: 34580) according to the manufacturer’s instructions. Protein bands were then visualized using the iBright CL1500 Imaging System (Invitrogen).

### 4.5. Cell proliferation and apoptosis assays

The Cell Counting Kit-8 (CCK-8, Sigma-Aldrich) was used to assess cell proliferation according to the manufacturer’s instructions. Cell suspensions (100 μl; 5 × 104 cells/ml) in medium containing 10% fetal bovine serum were seeded into 96-well plates and incubated at 37°C for various time periods (0, 24, 48, 72, and 96 hours). Subsequently, 10 μl of CCK-8 solution was added to each well, and the cells were incubated for an additional 2 hours at 37°C. The absorbance (OD) was then measured at 450 nm using using a Synergy HTX Multi-Mode Microplate Reader (BioTek Instruments, Inc.).

To determine the apoptosis rate, treated cells and controls were stained using an Annexin V-FITC/PI apoptosis detection kit (BD Biosciences) according to the manufacturer’s protocol. Following a 15-minute incubation with Annexin V-FITC/PI dye mixture in the dark at room temperature, cell apoptosis was analyzed using flow cytometry on a Beckman Coulter CytoFLEX Flow Cytometer (Beckman Coulter, Inc.). The percentage of apoptotic cells was calculated as the sum of early (Annexin V-FITC+/PI-) and late (Annexin V-FITC+/PI+) apoptotic cells.

### 4.6. Cell migration and invasion assays

Cell migration and invasion abilities were assessed using Transwell assays. For the invasion assay, 24-well Transwell inserts (8 μm pore size, Corning) were coated with Matrigel (1:8 dilution in serum-free medium), and the empty Transwell inserts without Matrigel were used for the migration assay. Cells (2 × 10^4) in serum-free medium were seeded in the upper chamber, with 10% FBS-containing medium in the lower chamber as a chemoattractant. After 24 hours of incubation, non-migrating/invading cells were removed, and the migrating/invading cells were fixed with 4% paraformaldehyde, stained with 0.1% crystal violet (Sigma-Aldrich), and counted in five random fields using an Olympus I×73inverted microscope. Both assays were performed in triplicate and repeated independently at least three times.

### 4.7. Cell transfection and shRNA-mediated knockdown

miRNA control (miR-NC) and miR-3940-5p mimic were synthesized by RibioBio (Guangzhou, China). The expression plasmid containing PDLIM1 cDNA, lentiviral vector for control shRNA (sh-NC), and sh-PDLIM1 were constructed by GenScript (Nanjing, China). For transient transfections, cells were seeded in 6-well plates at a density of 2 × 10^5 cells per well and cultured overnight. Transfections were performed using Lipofectamine 3000 (Invitrogen). The final concentrations of miRNA mimic and plasmids were 50 nM and 2 μg per well, respectively. Cells were harvested 48 hours post-transfection for subsequent experiments.

For lentiviral production, HEK293T cells were co-transfected with the lentiviral vector (sh-NC or sh-PDLIM1), psPAX2 (packaging plasmid), and pMD2.G (envelope plasmid) using Lipofectamine 3000. The culture medium containing lentiviral particles was collected 48 and 72 hours post-transfection, filtered through a 0.45 μm filter, and concentrated using PEG-it™ Virus Precipitation Solution (System Biosciences, Palo Alto, CA, USA) according to the manufacturer’s protocol. For lentiviral transduction, DLBCL cells (1 × 10^5 cells/ml) were infected with lentiviral particles at a multiplicity of infection (MOI) of 10 in the presence of 8 μg/ml polybrene (Sigma-Aldrich, St. Louis, MO, USA). After 24 hours, the medium was replaced with fresh complete medium. To establish stable cell lines, transduced cells were selected with puromycin (2 μg/ml) for 14 days. The knockdown efficiency was verified by qRT-PCR and western blot analysis.

### 4.8. Nude mouse model of xenograft tumor

Five-to-six-week-old male BALB/c nude mice were obtained from Shanghai SLAC Laboratory Animal Co., Ltd. (Shanghai, China). The mice were housed in a specific pathogen-free (SPF) environment under controlled conditions (temperature: 22 ± 2°C, humidity: 50 ± 10%, 12 h light/dark cycle) with ad libitum access to sterile food and water. After a one-week acclimatization period, SU-DHL-4 cells (1 × 10^7) stably transfected with sh-NC or sh-PDLIM1 were subcutaneously injected into the middle of the right armpit of nude mice. Tumor growth was monitored every three days, and tumor volume was calculated using the formula: V (mm^3) = π/6 × (Length) × (Width)^2, where length represents the longest diameter and width the shortest. 23 days post-inoculation, mice were euthanized by CO_2_ inhalation and tumor tissues were immediately excised, photographed, and weighed. Tumor tissues were then either snap-frozen in liquid nitrogen and stored at −80°C or fixed in 4% paraformaldehyde (PFA) for subsequent analyses. All animal procedures were approved by the Institutional Animal Care and Use Committee of Quanzhou First Hospital Affiliated to Fujian Medical University and were conducted in strict accordance with the National Institutes of Health Guide for the Care and Use of Laboratory Animals.

### 4.9. Hematoxylin and eosin staining

Tissue samples were subjected to a standard hematoxylin and eosin (H&E) staining protocol using an H&E Stain Kit (Abcam, Cambridge, UK). Briefly, the samples were hydrated in distilled water. Hematoxylin, Mayer’s (Lillie’s Modification) solution was applied to completely cover the tissue sections, followed by a 5-minute incubation. Excess stain was removed by rinsing twice with distilled water. Subsequently, Bluing Reagent was added to cover the tissue sections entirely and incubated for 15 seconds. Slides were then rinsed with distilled water and briefly dipped in absolute alcohol. Next, Eosin Y Solution (Modified Alcoholic) was applied to cover the tissue sections completely and incubated for 3 minutes. After incubation, slides were washed with absolute alcohol. Finally, the stained sections were dehydrated through a series of graded alcohols, cleared in xylene, and mounted with resin mounting medium.

### 4.10. Immunohistochemical analysis

Tissue samples were fixed in 10% neutral buffered formalin for 24 hours, dehydrated through a graded ethanol series, cleared in xylene, and embedded in paraffin. Sections (4 μm thick) were cut using a microtome and mounted on positively charged glass slides. For immunohistochemistry, sections were deparaffinized in xylene and rehydrated through graded ethanol to water. Antigen retrieval was performed using citrate buffer (pH 6.0) in a pressure cooker for 15 minutes. Endogenous peroxidase activity was quenched with 3% hydrogen peroxide for 10 minutes. Sections were then incubated overnight with primary antibody against Ki-67 (1:200 dilution; cat. no. A2094; ABclonal, Woburn, MA, USA) at 4°C. After washing three times with PBS-T (PBS containing 0.1% Tween-20), sections were incubated with HRP-labeled secondary antibody (1:500 dilution; cat. no. AS014; ABclonal) for 1 hour at room temperature. Following three more washes with PBS-T, sections were developed using 3,3’-diaminobenzidine (DAB) solution and counterstained with hematoxylin. Slides were then dehydrated, cleared, and mounted with coverslips using a permanent mounting medium. Ki-67 expression in cancer samples was analyzed using a light microscope (Olympus BX53, Tokyo, Japan) at 400× magnification.

### 4.11. Terminal deoxynucleotidyl transferase dUTP nick end labeling (TUNEL) assay

Apoptosis in tumor tissues was assessed using the In Situ Cell Death Detection Kit, POD (Roche Diagnostics, Basel, Switzerland) following the manufacturer’s protocol. Briefly, 5-μm paraffin-embedded tissue sections were deparaffinized, rehydrated, and permeabilized. After endogenous peroxidase blocking, sections were incubated with the TUNEL reaction mixture, followed by Converter-POD treatment. The signal was developed using DAB substrate and sections were counterstained with hematoxylin. Slides were dehydrated, cleared, and mounted. TUNEL-positive cells, exhibiting brown nuclear staining, were visualized using a light microscope (Olympus BX53, Tokyo, Japan).

### 4.12. Luciferase reporter assay

Constructs containing the wild-type PDLIM1 3′ UTR or its mutant (with the predicted miR-3940-5p binding site being mutated using the Q5® Site-Directed Mutagenesis Kit, New England Biolabs, Ipswich, MA, USA) were co-transfected into TMD8 and SU-DHL-4 cells along with miR-3940-5p mimic or a negative control (miR-NC) using Lipofectamine 3000 (Invitrogen, Thermo Fisher Scientific, Waltham, MA, USA). After 48 hours, cells were lysed and luciferase activities were measured using the Dual-Luciferase® Reporter Assay System (Promega, Madison, WI, USA) on a GloMax® Navigator Microplate Luminometer (Promega). Firefly luciferase activity was normalized to Renilla luciferase activity, which served as an internal control.

### 4.13. Statistical analyses

Data analysis was conducted using GraphPad Prism 8 (GraphPad Software, San Diego, CA, USA) and the results were expressed as mean and standard deviations of three independent experiments. Comparisons between two groups were made using unpaired two-tailed Student’s t-tests. For multiple group comparisons, one-way ANOVA followed by Tukey’s post hoc test was employed. Correlations between variables were assessed using Pearson’s correlation coefficient. Overall survival (OS) and disease-free survival (DFS) were analyzed using Kaplan-Meier curves, and differences between groups were evaluated using the log-rank test. A p-value <.05 was considered statistically significant for all analyses.

## Data Availability

The datasets generated during and/or analyzed during the current study are not publicly available, but are available from the corresponding author on reasonable request.

## References

[1] Tilly H, Gomes Da Silva M, Vitolo U, Jack A, Meignan M, Lopez-Guillermo A, Walewski J, André M, Johnson PW, Pfreundschuh M, et al. Diffuse large b-cell lymphoma (DLBCL): ESMO clinical practice guidelines for diagnosis, treatment and follow-up. Ann Oncol. 2015;26(Suppl 5):v116–11. doi:10.1093/annonc/mdv304. 26314773

[2] Sehn LH, Salles G, Longo DL. Diffuse large b-cell lymphoma. N Engl J Med. 2021;384(9):842–858. doi:10.1056/NEJMra2027612. 33657296 PMC8377611

[3] Zhou JK, Fan X, Cheng J, Liu W, Peng Y. PDLIM1: structure, function and implication in cancer. Cell Stress. 2021;5(8):119–127. doi:10.15698/cst2021.08.254. 34396044 PMC8335553

[4] Arthur SE, Jiang A, Grande BM, Alcaide M, Cojocaru R, Rushton CK, Mottok A, Hilton LK, Lat PK, Zhao EY, et al. Genome-wide discovery of somatic regulatory variants in diffuse large B-cell lymphoma. Nat Commun. 2018;9(1):4001. doi:10.1038/s41467-018-06354-3. 30275490 PMC6167379

[5] Chen HN, Yuan K, Xie N, Wang K, Huang Z, Chen Y, Dou Q, Wu M, Nice EC, Zhou Z-G, et al. PDLIM1 stabilizes the E-Cadherin/β-catenin complex to prevent epithelial–mesenchymal transition and metastatic potential of colorectal cancer cells. Cancer Res. 2016;76(5):1122–1134. doi:10.1158/0008-5472.CAN-15-1962. 26701804

[6] Huang Z, Zhou JK, Wang K, Chen H, Qin S, Liu J, Luo M, Chen Y, Jiang J, Zhou L, et al. PDLIM1 inhibits tumor metastasis through activating hippo signaling in hepatocellular carcinoma. Hepatol. 2020;71(5):1643–1659. doi:10.1002/hep.30930. 31509262

[7] Qiu C, Duan Y, Wang B, Shi J, Wang P, Ye H, Dai L, Zhang J, Wang X. Serum anti-PDLIM1 autoantibody as diagnostic marker in ovarian cancer. Front Immunol. 2021;12:698312. doi:10.3389/fimmu.2021.698312. 34489945 PMC8417125

[8] Li LM, Luo FJ, Song X. MicroRNA-370-3p inhibits cell proliferation and induces chronic myelogenous leukaemia cell apoptosis by suppressing PDLIM1/Wnt/β-catenin signalling. Neoplasma. 2020;67(3):509–518. doi:10.4149/neo_2020_190612N506. 31986893

[9] O’brien J, Hayder H, Zayed Y, Zayed Y, Peng C. Overview of MicroRNA biogenesis, mechanisms of actions, and circulation. Front Endocrinol. 2018;9:402. doi:10.3389/fendo.2018.00402. PMC6085463 30123182

[10] Di Leva G, Garofalo M, Croce CM. MicroRNAs in cancer. Annu Rev Pathol Mech Dis. 2014;9(1):287–314. doi:10.1146/annurev-pathol-012513-104715. PMC4009396 24079833

[11] Yousef M, Goy G, Bakir-Gungor B. miRModuleNet: detecting miRNA-mRNA regulatory modules. Front Genet. 2022;13:767455. doi:10.3389/fgene.2022.767455. 35495139 PMC9039401

[12] Tang Z, Li C, Kang B, Gao G, Li C, Zhang Z. GEPIA: a web server for cancer and normal gene expression profiling and interactive analyses. Nucleic Acids Res. 2017;45(W1):W98–W102. doi:10.1093/nar/gkx247. 28407145 PMC5570223

[13] Tomczak K, Czerwińska P, Wiznerowicz M. The cancer genome atlas (TCGA): an immeasurable source of knowledge. Contemp Oncol (Pozn). 2015;1A(1):68–77. doi:10.5114/wo.2014.47136. PMC4322527 25691825

[14] Xu-Monette ZY, Tu M, Jabbar KJ, Cao X, Tzankov A, Visco C, Cai Q, Montes-Moreno S, An Y, Dybkaer K, et al. Clinical and biological significance of de novo CD5+ diffuse large B-cell lymphoma in Western countries. Oncotarget. 2015;6(8):5615–5633. doi:10.18632/oncotarget.3479. 25760242 PMC4467390

[15] Garzon R, Calin GA, Croce CM. MicroRNAs in cancer. Annu Rev Med. 2009;60(1):167–179. doi:10.1146/annurev.med.59.053006.104707. 19630570

[16] Alsaadi M, Khan MY, Dalhat MH, Bahashwan S, Khan MU, Albar A, Almehdar H, Qadri I. Dysregulation of miRNAs in DLBCL: causative factor for pathogenesis, diagnosis and prognosis. Diagn (Basel). 2021;11(10):1739. doi:10.3390/diagnostics11101739. PMC8535125 34679437

[17] Shi Y, Ding D, Qu R, Tang Y, Hao S. Non-coding RNAs in diffuse large B-cell lymphoma. OTT. 2020;13:12097–12112. doi:10.2147/OTT.S281810. PMC7699984 33262609

[18] Lujambio A, Lowe SW. The microcosmos of cancer. Nature. 2012;482(7385):347–355. doi:10.1038/nature10888. 22337054 PMC3509753

